# Performance of Current Chronic Kidney Disease Screening Criteria in Women and Men Across Ethnic Groups: The HELIUS Study

**DOI:** 10.1016/j.mayocpiqo.2025.100613

**Published:** 2025-04-17

**Authors:** Taryn G. Vosters, Vianda S. Stel, Kitty J. Jager, Bart Ferwerda, Roos F. Marsman, Frans J. van Ittersum, Bert-Jan H. van den Born, Henrike Galenkamp, Liffert Vogt, Irene G.M. van Valkengoed

**Affiliations:** a Department of Public and Occupational Health, Amsterdam Public Health Research Institute, Amsterdam UMC, University of Amsterdam, Amsterdam, Netherlands; b Department of Medical Informatics, Amsterdam Public Health Research Institute, Amsterdam UMC, University of Amsterdam, Amsterdam, Netherlands; c Department of Internal and Vascular Medicine, Amsterdam Public Health Research Institute, Amsterdam UMC, University of Amsterdam, Amsterdam, Netherlands; d Amsterdam Public Health Research Institute, Quality of Care and Ageing Later Life, Amsterdam, Netherlands; e Department of Clinical Epidemiology, Biostatics and Bioinformatics, Amsterdam, Amsterdam UMC, University of Amsterdam, Netherlands; f Section Nephrology, Department of Internal Medicine, Amsterdam Cardiovascular Sciences, Amsterdam, Amsterdam UMC, University of Amsterdam, Netherlands; g Section Nephrology, Department of Internal Medicine, Amsterdam Cardiovascular Sciences, Amsterdam UMC, VU University, Amsterdam, Netherlands

## Abstract

**Objective:**

To investigate whether the currently recommended screening criteria in Kidney Disease: Improving Global Outcomes 2024 guidelines (hypertension, diabetes mellitus, and cardiovascular disease) equally detect women and men across ethnic groups and whether consideration of optional criteria (education level, occupation, obesity, and genetic risk factors) listed in the guideline improves performance.

**Patients and Methods:**

We included 12,384 women and 9046 men of Dutch, South Asian and African Surinamese, Ghanaian, Turkish, and Moroccan origin from the baseline HELIUS Study (January 1, 2011, through December 31, 2015, Amsterdam, the Netherlands). Chronic kidney disease (CKD) was defined as estimated glomerular filtration rate of <60 mL/min/1.73 m^2^ or albumin-to-creatinine ratio of >3 mg/mmol. Poisson regression analyses estimated associations between CKD and optional criteria on top of current screening criteria. Model comparisons were made with likelihood ratio tests and Akaike information criterion estimations in women and men. Area under the curve (AUC), sensitivity, specificity, and positive and negative predictive values were calculated by sex and ethnicity.

**Results:**

Chronic kidney disease prevalence ranged from 2.9% to 8.8% in women and 3.2% to 8.6% in men. Low educational level (women only) and obesity significantly improved the models with current criteria with CKD. High-risk occupations and polygenic risk score did not improve the model. However, these criteria did not improve predictive measures across ethnic groups. Overall, the AUCs for the current screening criteria were acceptable in men (AUC, 0.75; 95% CI, 0.73-0.77) and poor in women (AUC, 0.65; 95% CI, 0.63-0.67), and showed minimal change after adding the optional criteria.

**Conclusion:**

Current screening criteria may not be equally detecting women and men across ethnic groups with CKD. Optional criteria had limited added value.

Chronic kidney disease (CKD) is a leading public health problem, with the global prevalence on the rise.^1^ Women tend to have higher prevalence rates of CKD than men (14.6% vs 12.8%),^1^ whereas men in most populations have a faster progression of the disease.^2^^,^^3^ Additionally, the burden falls disproportionally on socially disadvantaged population groups, including ethnic minority groups living in high-income countries.

Early CKD detection allows for a faster implementation of treatment, which can prevent or slowdown the progression of CKD and the associated complications such as end-stage renal failure, as well as the need for kidney replacement therapy.^4^ However, the early stages of CKD follow an asymptomatic trend for most patients. Therefore, case finding in populations at risk is important to improve patient outcomes and reduce the impact CKD has on health care resources worldwide.

The Kidney Disease: Improving Global Outcomes (KDIGO) guidelines and controversies conference reports^5^^,^^6^ formulate criteria that may be used to identify those eligible for CKD screening. They state that the presence of hypertension, diabetes, cardiovascular disease (CVD), a family history of CKD, or a history of acute kidney injury (AKI) indicate a patient should be screened for CKD. Further optional medical risk factors are mentioned in the guidelines: for example, social risk factors such as low socioeconomic status (SES), obesity, genetic risk factors, and high-risk occupations. However, the value of these criteria has not been evaluated by sex or in a multiethnic population. It is unknown whether the current screening CKD criteria may miss subgroups with prevalent early-stage CKD or if the optional criteria mitigate this. Differences between subgroups are likely because previous research has shown that clinical risk factors, such as hypertension and diabetes, are more prevalent in men than that in women across ethnic groups.^7^ Differing distribution of risk factors across women and men may influence detection.^8^ Recent evaluation of screening guidelines for CVD, a disease with a shared mechanism and risk factors with CKD,^9^ revealed women as well as ethnic groups, such as Surinamese, Ghanaian, Turkish, and Moroccan groups, at high-risk of CVD are less frequently identified based on the given criteria.^10^

The aim of our study was to evaluate the performance of the current KDIGO guidelines for CKD screening in women and men across ethnic groups. First, we aimed to determine the predictive value of the currently recommended CKD screening criteria from the guideline (hypertension, diabetes, and CVD) in women and men aged 18-70 years, from a multiethnic population living in the Netherlands. Second, we aimed to determine to what extent optional screening criteria (low education as a marker of SES, obesity, genetic risk factors, and high-risk occupations) add to CKD detection on top of the currently recommended criteria.

## Patients and Methods

### Study Design and Study Population

We used baseline data from the prospective population-based Healthy Life in an Urban Setting (HELIUS) study,^11^ collected between January 1, 2011, and December 31, 2015, in men and women from 6 different ethnic groups including Dutch, Surinamese (South Asian and African), Ghanaian, Turkish and Moroccan, aged between 18 and 70 years and living in Amsterdam. Participants were randomly sampled from the municipality registry, stratified by ethnicity based on their registered country of birth and that of their parents. Data were obtained via questionnaire and physical examinations (including biological samples), before which participants were asked whether they had a fever or experiencing any acute illnesses. The study has been previously described in further detail.^11^ Ethical approval was given by the Ethical Review Board of the Academic Medical Centre Amsterdam. All participants provided written informed consent.

Of the 22,162 individuals with questionnaire and physical examination data, we excluded those of Javanese Surinamese or other/unknown Surinamese origin because of their low group size, excluding a sum of 548 participants). Additionally those without estimated glomerular filtration rate (eGFR) and albumin-to-creatinine ratio (ACR) measurements were excluded (n=184). The final sample consisted of 21,430 participants of which 9046 were men and 12,384 were women.

### Chronic Kidney Disease

The CKD was defined based on ACR and the eGFR values from a single sample (Table 1).^12^, ^13^, ^14^, ^15^, ^16^, ^17^, ^18^, ^19^ CKD was considered present for those in the moderately increased, high-risk, and very high-risk categories according to the KDIGO guidelines (eGFR <60 mL/min/1.73 m^2^ and/or ACR ≥3 mg/mmol).

**Table 1:** Included Variable Definitions

Variable	Measurement	Definition
Kidney function estimates		
eGFR	GFR was estimated with the Chronic Kidney Disease Epidemiology (CKD-EPI 2021) equation using creatinine, which does not include a correction factor for ethnicity. Serum creatinine concentration, from a single fasting blood sample taken during physical examination, was determined in a single measurement by a kinetic colorimetric spectrophotometric method (Roche Diagnostics).	eGFR < 60 mL/min/1.73 m^2^
ACR	Albuminuria was assessed by ACR in a single morning urine sample. Urine creatinine and ACR were analyzed by kinetic spectrophotometric and immune chemical turbidimetric methods, respectively (both Roche Diagnostics)	ACR ≥ 3 mg/mmol
CKD	Risk categories based on eGFR and ACR measurements using KDIGO cutoffs	eGFR < 60 mL/min/1.73 m^2^ and/or ACR ≥ 3 mg/mmol
KDIGO current screening criteria		
Hypertension	BP was measured twice on the left arm, in a seated position after 5 min of rest during the physical examination using a semiautomated oscillometric device. The average of both measurements was recorded	Defined by values ≥140 mm Hg systolic BP and/or ≥90 mm Hg diastolic BP, or being on BP-lowering medication (angiotensin-converting enzyme inhibitors, angiotensin II receptor blockers, diuretics, or β-blockers at the time of data collection)
Diabetes mellitus	Fasting blood samples were taken during physical examination, and glucose was determined using an enzymatic spectrophotometric method (Roche Diagnostics). Diabetes was also self-reported via questionnaire	Defined as self-reported diabetes, fasting glucose of ≥7 mmol/L in a single measurement and/or use of antidiabetic agents (biguanides, sulfonylureas, DPP-4 inhibitors, SGLT2 inhibitors, or insulin)
Cardiovascular disease	Participants stated ever having an adverse cardiac event in the questionnaire. Information on the use of secondary preventive medication was collected during the physical examination	Defined if participants stated ever having had 1 or more of the following: stroke/heart attack/bypass or stent surgery
Family history of CKD	Data were not available in this study	
History of acute kidney injury	Data were not available in this study	
KDIGO optional screening criteria		
Low socioeconomic status	Assessed based on the educational level of the participant, as for the Netherlands, this has been proven to be more stable than an income-based variable.^12^ Educational level was self-reported via questionnaire and then categorized into low (never been to school or elementary school), low-middle (lower vocational schooling or lower secondary school), middle-high (intermediate vocational schooling or higher secondary schooling), and high (higher vocational schooling or university)	Defined as those stating never having been to school or only completing elementary school
Obesity	Weight measured during physical examination, in light clothing on a Seca877 scale and height measured without shoes using a Seca217 stadiometer	Defined as a body mass index of ≥30 kg/m^2^
Genetic risk factors	Operationalized by polygenic risk score (PRS), based on current evidence.^13^ A total of 147 genome-wide significant genetic variants associated with CKD were selected from a previously validated transancestry genome-wide association study (GWAS) (Supplementary Material, available online at http://www.mcpiqojournal.org).^13^ The associated GWAS variations were selected within the genotyped HELIUS subgroup of individuals across all included ethnic groups (n=10,284), and PRS was calculated using the PLINK software to estimate the individual risk of CKD using the protocol descripted in Choi et al.^14^^,^^15^ This score is a sum of the GWAS-associated variants an individual carries and represents it genetic risk of CKD in relation to the population. Scaling was done for women and men separately (Supplemental Figure 1)	The risk scores were divided into quintiles; the highest quintile was considered high risk and the rest as low risk. Scaling and subsequent analyses conducted using the PRS were not further stratified by ethnicity owing to small numbers
High-risk occupations	Participants stated their current or former occupation via questionnaire	High-risk occupations were based on literature, including agricultural, and construction workers,^16^^,^^17^ grouped using the occupational classification from the Statistics Netherlands,^18^ and further categorized into high-risk or low risk occupation. Those who did not work were categorized as nonapplicable and fell under low risk such as students, those unable to work, pensioners, and those receiving social benefits
Poor geographical access to health care	Perceived as less relevant in study setting considering all participants live in Amsterdam, where the average distance to the nearest primary care facility is 1 km and hospital is <5 km^19^	
HIV	Data were not available in this study	
Lupus	Data were not available in this study	
Other variables		
Sex	Derived from municipal registry	Woman/man
Age	Derived from municipal registry	Years
Ethnicity	Determined by the registered country of birth of the participant and their parents. Surinamese participants were further categorized after data collection into South Asian Surinamese and African Surinamese, Javanese, or other, based on self-report.	Participant was defined as of non-Dutch origin when 1 of the following criteria was met: they were born outside of the Netherlands or at least 1 parent was born outside of the Netherlands. Dutch participants were selected if both the participant and both parents were born in the Netherlands.

ACR, albumin-to-creatinine ratio; BP, blood pressure; CKD, chronic kidney disease; GFR, glomerular filtration rate; eGFR, estimated glomerular filtration rate; KDIGO, kidney disease improving global outcomes.

### KDIGO Screening Criteria

The KDIGO guidelines recommend a patient to be screened for CKD when the following is present: hypertension, diabetes mellitus, CVD, a family history of CKD, and a patient history of AKI. Owing to the unavailability of information, we could not include family history of CKD and history of AKI. Eligibility (yes/no) was therefore defined as presence of hypertension or diabetes mellitus or CVD. Criteria such as low SES, obesity, genetic risk factors, and high-risk occupations with high exposure to nephrotoxins are recommended by KDIGO as optional factors to identify CKD in patients. Other optional variables were perceived as less relevant in the Dutch context, that is, poor geographical access to health care, or were unavailable, namely information on HIV status and lupus.

### Statistical Analyses

Baseline characteristics in women and men across ethnic groups were presented by their mean (SD), median (IQR), or frequencies (percentages). The distribution was examined visually (histograms) to determine normality.

We used Poisson regression analyses to determine the association of the current criteria for screening with prevalent CKD by adding the variables to the model separately in women and men. This model is referred to as the base model, including hypertension, diabetes mellitus, and CVD. Then, we determined the value of adding separate optional variables to the base model in women and in men. For low education and obesity, this was done in the full population, for high-risk occupation in those who work and for the polygenic risk score (PRS) for those who had whole genome sequencing available. Whether these factors improved the predictive value of the base model in women and men was assessed by likelihood ratio tests comparing the model with the optional variable to the base model within the same subset of the population. Because the addition of the optional criteria were expected to generally increase the explained variance, we reported the Akaike information criterion (AIC) to indicate a more accurate, and thus better fitting model. We then stratified these analyses by ethnicity to verify the consistency of our findings across groups. The final model was derived by backward stepwise elimination of the variables that significantly improved the base model in women and men in the overall population, in the order of lowest to highest AIC.

Whether there was an improvement in discriminative ability between the base and final model in women and men and the base and final model in women and men across ethnic groups was assessed by comparison of the area under the curve (AUC) of the receiver operator characteristics and 95% CIs by bootstrapping calculated with the R package *pROC*. Common cutoff values were used to interpret the AUC values: 0.5-0.6, no discrimination; greater than or equal to 0.6 to less than 0.7, poor; greater than or equal to 0.7 to less than 0.8, acceptable; greater than or equal to 0.8 to less than 0.9; excellent; and greater than or equal to 0.9, outstanding.^20^ Other predictive measures such as sensitivity, specificity, and positive and negative predictive values were also calculated at the optimal threshold. Optimal thresholds were estimated by minimizing the difference between the true positive and false positive rates.

We further repeated the association analyses with a combined variable identifying all cases with hypertension and/or diabetes and/or CVD as eligible (yes/no), as stated in the guideline to reflect daily practice. Additionally, we estimated the predictive measures of this approach. Finally, we repeated the association analyses for high-risk occupation in a subset including only individuals that stated to currently be working. Analyses were considered significant at *P*<.05, and all statistical analyses were conducted in R-Studio 4.2.1.

## Results

The overall mean age in men was 44.9 (13.2) years, and in women, it was 43.7 (13.2) years. Women had higher proportion of low education compared with men (20.5% vs 14.1%) (Table 2). Working in a high-risk occupation was more common in men than that in women. This was more prevalent across ethnic groups, specifically in the Ghanaian participants, in comparison with the Dutch reference group. Clinical risk factors including hypertension, diabetes mellitus, and CVD were more prevalent overall in men, whereas obesity was more prevalent in women. Overall, screening eligibility based on current CKD criteria varied from 26.4% to 66.7% across all groups. Supplemental Table 1 shows baseline characteristics of the ACR subset.

**Table 2:** Eligibility for CKD Screening by CKD Status

	CKD: yes	CKD: no
Women		
Base criteria: yes	66.3	41.9
Base criteria: no	33.7	58.1
Men		
Base criteria: yes	84.2	46.4
Base criteria: no	15.8	53.6

Base criteria refers to the presence of hypertension, diabetes, and cardiovascular disease; CKD is defined as eGFR <60 mL/min/1.73 m^2^ and/or ACR >3 mg/mmol.

ACR, albumin-to-creatinine ratio; CKD, chronic kidney disease; eGFR, estimated glomerular filtration rate.

Overall CKD prevalence was slightly higher in women than that in men: 6.3% and 5.5%, respectively. Sex differences in prevalence varied across ethnic groups, with Ghanaian, Turkish, and Moroccan origin men having lower prevalences and Dutch and South Asian and African Surinamese men having equal or somewhat higher prevalences than women of the same origin.

A crosstabulation between those who qualify for CKD screening based on the current CKD criteria (base criteria) and those with CKD showed that a higher percentage of men were accurately detected than women (84.2% vs 66.3%) (Table 3).

**Table 3:** Baseline Characteristics of Women and Men Overall and Across Ethnic Groups

Variables	Women							Men						
	Overall (n=12,384)	Dutch (n=2456)	SA Surinamese (n=1663)	African Surinamese (n=2506)	Ghanaian (n=1417)	Turkish (n=1961)	Moroccan (n=2381)	Overall (n=9046)	Dutch (n=2069)	SA Surinamese (n=1363)	African Surinamese (n=1599)	Ghanaian (n=896)	Turkish (n=1617)	Moroccan (n=1502)
Mean age (y)	43.77 (13.2)	45.55 (14.2)	46.09 (13.2)	47.81 (12.3)	43.36 (10.7)	39.91 (12.2)	39.46 (12.9)	44.93 (13.2)	46.91 (13.8)	44.84 (13.6)	48.11 (12.9)	46.89 (11.4)	40.89 (12.2)	42.07 (12.76)
Educational level (missing n=193)														
Low	20.5	3.2	15.7	5.0	36.8	37.3	34.7	14.1	3.4	12.9	6.6	16.1	24.8	25.6
Low-medium	24.4	14.8	34.1	32.7	36.3	20.0	15.4	28.8	13.6	32.4	40.7	45.6	30.6	21.9
Medium high	28.7	20.6	27.9	36.5	22.3	28.7	33.3	29.6	23.5	30.7	34.1	29.4	28.6	33.4
High	26.3	61.3	22.3	25.8	4.5	14.0	16.6	27.5	59.6	23.9	18.6	8.9	16.0	19.1
Occupational risk														
Low risk	97.9	99.8	98.2	98.6	93.0	95.4	99.5	91.5	95.0	92.2	89.2	90.6	88.0	91.9
High risk	2.1	0.2	1.8	1.4	7.0	4.6	0.5	8.5	5.0	7.8	10.8	9.4	12.0	8.1
BMI														
Normal	37.6	65.8	41.8	28.5	18.3	30.1	32.9	40.2	53.4	45.5	41.6	35.1	25.0	35.1
Overweight	31.3	24.0	34.8	33.9	37.3	29.1	31.9	42.5	36.6	40.8	41.2	47.3	46.9	45.6
Obese	31.1	10.2	23.5	37.6	44.4	40.9	35.2	17.4	10.1	13.7	17.2	17.7	28.1	19.3
Hypertension (missing n=47)														
Yes	34.6	23.5	40.5	50.7	52.3	26.3	21.2	41.1	37.1	45.0	50.1	62.4	32.9	29.6
No	65.4	76.5	59.5	49.3	47.7	73.7	78.8	58.9	62.9	55.0	49.9	37.6	67.1	70.6
Diabetes														
Yes	10.0	2.4	17.7	12.1	9.7	9.3	10.9	12.0	5.1	21.6	11.7	14.8	11.3	12.3
No	90.0	97.6	82.3	87.9	90.3	90.7	89.1	88.0	94.9	78.4	88.3	85.2	88.7	87.7
Genetic risk factors^a^^,^^b^														
Yes	1124 (20.0)							864 (20.0)						
No	4500 (80.0)							3459 (80.0)						
CVD (missing n=311)														
Yes	4.1	2.6	6.0	4.2	3.8	6.0	2.7	7.0	4.9	13.0	6.9	5.9	7.5	4.5
No	95.9	97.4	94.0	95.8	96.2	94.0	97.3	93.0	95.1	87.0	93.1	94.1	92.5	95.5
Eligibility criteria^c^														
Yes	43.4	26.4	50.9	57.7	58.1	41.4	33.9	48.5	40.3	54.9	55.1	66.7	43.9	41.3
No	56.6	73.6	49.1	42.3	41.9	58.6	66.1	51.5	59.7	45.1	44.9	33.3	56.1	58.7
Median eGFR (mL/min/1.73 m^2^)	104.4 (91.2-114.6)	97.9 (85.7-107.2)	103.3 (92.6-112.0)	94.4 (82.3-105.1)	97.5 (84.6-107.9)	112.4 (105.0-120.7)	115.3 (107.0-123.6)	101.7 (89.5-111.5)	101.0 (90.7-110.5)	100.0 (88.1-109.6)	91.7 (80.5-102.5)	89.6 (79.1-100.5)	108.8 (100.6-117.2)	109.1 (101.1-117.5)
Median ACR (mg/mmol)	0.31 (0.19-0.60)	0.27 (0.17-0.42)	0.31 (0.19-0.63)	0.29 (0.18-0.57)	0.28 (0.18-0.60)	0.37 (0.22-0.75)	0.40 (0.23-0.81)	0.23 (0.14-0.44)	0.22 (0.14-0.37)	0.25 (0.14-0.55)	0.22 (0.13-0.45)	0.20 (0.12-0.42)	0.25 (0.15-0.46)	0.26 (0.16-0.49)
CKD prevalence^d^	6.3	2.9	7.3	6.7	8.8	7.1	6.6	5.6	3.2	8.6	6.6	8.3	5.0	4.1

Data are presented as percentages unless specified.

ACR, albumin-to-creatinine ratio; BMI, body mass index; CVD, cardiovascular disease; eGFR, estimated glomerular filtration rate; SA, South Asian.

a Based on the calculation of polygenic risk score with threshold being the highest quintile.

b Analyses conducted in a subgroup of the cohort where data from whole genome sequencing were available. Owing to small numbers, these were not stratified by ethnicity.

c The presence of hypertension or diabetes or CVD.

d Defined as eGFR <60 mL/min/1.73 m^2^ and/or ACR of >3 mg/mmol.

**Figure 1:**
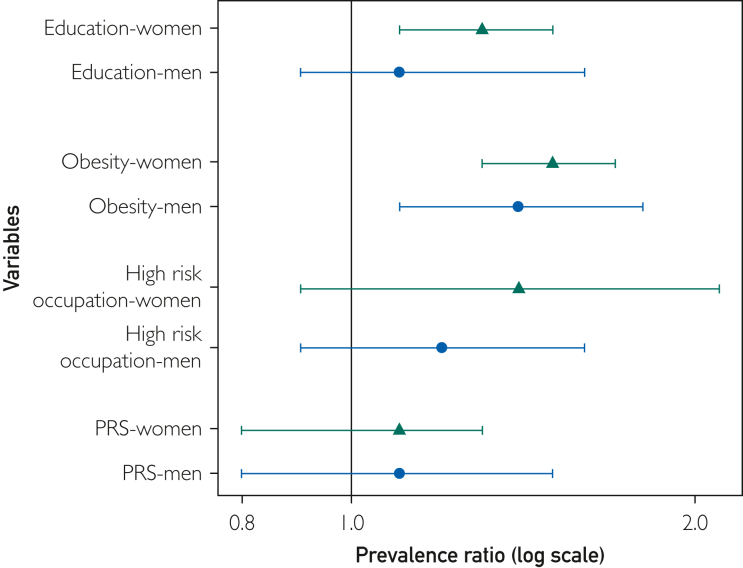
Prevalence ratios for chronic kidney disease of the optional variables adjusted for current screening criteria in women and men overall. Base model included hypertension, diabetes mellitus, and cardiovascular disease. PRS, polygenic risk score.

Adding low educational level and obesity to the current criteria statistically significantly improved the model in women (Figure 1) (Supplemental Table 1, available online at http://www.mcpiqojournal.org). For men, a statistically significant improvement was only observed when adding obesity to the current criteria (likelihood ratio tests, *P*=.002), but not for low education. High-risk occupations and PRS did not significantly improve the models for either women or men. The patterns were not consistent across ethnic groups. The final model contained the current screening criteria and obesity in men and women and additionally including low education for women (Supplemental Table 2, available online at http://www.mcpiqojournal.org). The AIC of the final models was lower compared with the base model (AIC, 4253.2 vs 4273.0 in women and 3412.0 vs 3419.6 in men).

**Figure 2:**
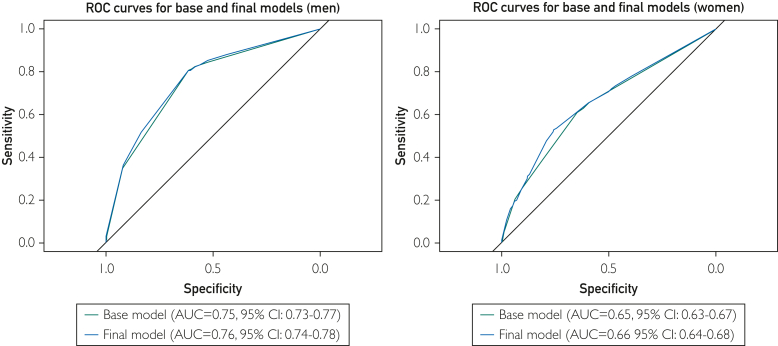
Base and final models of AUC comparison. Base model included hypertension, diabetes, and cardiovascular disease; final model included base model + education and obesity (women only). AUC, area under the receiver operator characteristic curve; ROC, receiver operator characteristic.

The AUCs for the base model were acceptable in men and poor in women (AUC, 0.75; 95% CI, 0.73-0.77, in men; AUC, 0.64; 95% CI, 0.62-0.66, in women) (Figure 2) (Supplemental Table 3, available online at http://www.mcpiqojournal.org). The poor performance in women was observed in all ethnic groups, and in men, we observed a broader range from poor to excellent (range AUC, 0.57-0.68 in women vs 0.65-0.81 in men). For the final model, the AUCs and patterns of differences between women and men was not substantially different for most groups. This lack of change is reflected in the overall lack of improvement in other parameters.

Consideration of the alternative classification reflecting daily practice (eligibility based on either hypertension and/or diabetes and/or CVD) and the analysis only including the working population gave similar results to our main analyses (Supplemental Table 4, available online at http://www.mcpiqojournal.org). As in the main analyses, both models showed higher AUC, better sensitivity and similar specificity detection in men in comparison with those in women. A lack of improvement in AUC between the base and final models was observed (Supplemental Table 5, available online at http://www.mcpiqojournal.org).

## Discussion

### Main Findings

This study aimed to evaluate the performance of the current CKD screening guidelines in women and men and across ethnic groups. Our results show that the current CKD screening criteria (ie, hypertension, diabetes, and CVD) performed better in men than those in women overall as well as across ethnic groups. Additionally, this study showed that some optional criteria, specifically low education (women only) and obesity, were associated with CKD on top of current criteria but did not substantially improve the ability to predict CKD in any of the groups.

### Limitations

Certain limitations should be considered before the interpretation of our results. First, we were not able to include all criteria mentioned in the KDIGO guideline because our data did not include variables such as a family history of CKD and a history of AKI. Thus, our results may not reflect the full performance of the guideline recommended screening criteria, and this study should be repeated using all recommended criteria in a clinical setting.

Moreover, we used self-reported and epidemiologic definitions of the screening eligibility criteria, which—although they would reflect practice—may have led to misclassification. For instance, participants who no longer worked did not answer all occupational questions in the questionnaire, although they were instructed to do so, which prevented us from classifying them as having worked in a high-risk occupation or not.

Our definition of prevalent CKD was based on a single sample from a general population, being commonly used in epidemiologic studies. However, clinical practice recommends using multiple samples to confirm chronicity and rule out AKI.^21^ Additionally, we used serum creatinine to estimate glomerular filtration rate, which may experience certain biases such as muscle mass and dietary consumption. An alternative method would be to estimate glomerular filtration rate using cystatin C measurements because these are not influenced by the abovementioned factors; however, these were not available.

### Discussion of Main Findings

Our study showed that current screening criteria missed a substantial proportion of those with CKD. This is reflected in the performance of the AUCs that were acceptable in men and poor in women. A suboptimal CKD detection ability may relate to choice of eligibility criteria from an international guideline; in our study, the selection was based on the 2024 KDIGO guideline, which being intended to be globally applicable. However, other national guidelines such as the Netherlands’ CVRM guideline, the UKs NICE guideline, or the European Renal Association best practice guideline generally recommend similar risk factors for CKD screening.^22^^,^^23^ Slight variations between recommendations exist, such as NICE includes the presence of structural renal tract disease as well as gout as a risk factor for CKD, whereas the US National Kidney Foundation practice guideline includes a focus on older age.^24^ Thus, the findings reflect a more generalized inability to detect CKD in a general population.

Importantly, the current criteria applied for CKD screening perform better in men than that in women in our study, and this pattern was consistent across ethnic groups. To our knowledge, sex differences in the performance of screening criteria have not been investigated. However, our results are in line with an evaluation of similar screening criteria for the identification of high 10-year risk of CVD.^10^ The sex differences also align with our previous finding that men tend to have a higher contribution of traditional risk factors to the population CKD prevalence than women.^7^ Others have argued that if men are more likely to have one of the current CKD criteria, CKD may more likely be detected when using the current CKD screening criteria in comparison to women.^8^ Another study confirmed Swedish women were less likely to receive a CKD diagnosis and referral to a nephrologist based on the clinical screening criteria.^25^

Considering that women globally have a higher prevalence of CKD than men when using current eGFR formulas, it is important to ensure the methods being used are as effective at detecting risk of disease in women as in men. We showed that this cannot be achieved by considering any of the evaluated optional criteria for screening. Although in line with previous work,^26^ we found an association of obesity with CKD, we did not observe an improvement in CKD detection. A possible explanation could be that obesity is largely associated with CKD through its association with the current screening criteria, and therefore, the current eligibility criteria already capture the excess risk.

Moreover, whereas literature shows women tend to be more affected by adverse sociocultural factors such as low SES than men,^27^ low education did not substantially improve prediction in our study. This aligns with a study conducted in the United States showing that adding neighborhood SES, which also included educational level, to the current criteria did not improve CKD prediction/detection in the general population.^28^ Contrastingly, the Dutch PREVEND study found that adding educational level to the current criteria did lead to detection of a larger group of individuals with a high-risk of CKD.^29^ The authors hypothesized that the higher rate of smokers in the selected educational groups in their study may explain their results. The researchers, however, did not stratify for sex, nor was a multiethnic population used. This is relevant as socioeconomic gradients in smoking differ by ethnicity/sex, also in the Netherlands.^30^

Our findings also showed no significant role for high-risk occupation for determining eligibility on top of current screening criteria. The lack of association may be explained by the low prevalence of individuals in our cohort working in high-risk occupations such as agricultural work and metal processing. This may relate to the urban setting, which, for instance, has no agricultural work opportunities, from which our study population was selected from. It is also possible that the work circumstances and climate influences in the Netherlands differ from those in other countries. For instance, studies that have shown agricultural work to have a negative effect on workers’ health were generally done in warmer climates with polluted sources of drinking water.^17^

Finally, we did not observe a possible contribution of genetic factors to prediction of CKD on top of the current criteria. This may relate to the PRS, the measure used to operationalize genetic risk, in general performing poorly. Nevertheless, the scores are in line with another study that investigated the association between PRS and CKD prevalence across multiple ancestries, which found that their PRS explained only 4% of the variance.^31^ Simultaneously, we recognize that we may have underestimated the predictive value in certain ethnic groups. The choice of genetic single nucleotide polymorphisms used within the subset is limited to 1 genome-wide association study (GWAS) and may not have been appropriate for the multiethnic and admixture nature of our sampled population. However, because there is limited literature in diverse populations and no definitive genetic risk factors listed in the guideline, we relied on literature from the CKDGen Consortium.^13^^,^^32^ Additionally, the PRS’s were not scaled per ethnicity due to low power and did not include the *APOL1* gene variant, which is a known risk factor CKD in populations of African descent.^33^ *APOL1* was not included in the selected GWAS. More GWAS need to be conducted including more ethnically diverse populations to increase the evidence for these groups. However, critical reflection is necessary. Although genetic testing offers advantages in targeted interventions and precise diagnostics, PRS calculations may be difficult to translate into clinical practice currently given the high costs and other services such as genetic counseling.

Not detecting a substantial proportion of women and men in a potentially high-risk multiethnic population using the KDIGO guidelines, considering the already existing disparities in CKD burden, has the implication of potentially increasing health inequalities. Detecting disease at a late stage may increase the risk of mortality.^8^ We recommend to increase awareness of unequal detection ability between women and men in certain contexts. Additionally, if similar disparities in detection rates are confirmed in other populations and settings, we would recommend to perhaps modify the list of optional criteria to increase detection of CKD. Finally, to ensure a more effective disease risk detection, particularly in women, additional sex-specific variables may be considered to specifically predict risk. Research has also shown that sex-specific characteristics may influence disease risk, such as pregnancy, lifetime duration of estrogen exposure, and menopause.^34^

An alternative to high-risk group screening is regular population wide screening. Although this may be effective in theory, in practice, it comes with many financial and labor-intensive barriers, which may hinder its feasibility.^35^

## Conclusion

In summary, the current screening criteria on hypertension, diabetes mellitus, and CVD according to the KDIGO guidelines are moderate to acceptable in identifying CKD, and the performance was better in men than women across ethnic groups. Additionally considering several optional criteria listed in the current KDIGO guidelines, including low education, obesity, high-risk occupations, and genetic risk factors, does not improve CKD prediction in either women or men across ethnic groups.

## Potential Competing Interests

Dr Stel reports institutional grants from the European Renal Association. Dr Jager reports registry funding from the European Renal Association and European Society for Paediatric Nephrology. Dr Vogt reports honorarium payments made to the institution from AstraZeneca, Bayer, Boehringer Ingelheim, CSL Vifor, Medice, and Sanofi Genzyme. The other authors have no competing interests to report.

## Ethics Statement

The HELIUS Study has been approved by the AMC Ethical Review Board (MREC 10/100# 17.10.1729). All participants provided written informed consent.
