# Supplementary material for: Performance of Current Chronic Kidney Disease Screening Criteria in Women and Men Across Ethnic Groups: The HELIUS Study
Source: Mayo Clin Proc Innov Qual Outcomes. 2025 Apr 17;9(3):100613. doi: 10.1016/j.mayocpiqo.2025.100613 (PMC12033983; doi:10.1016/j.mayocpiqo.2025.100613)
Supplement: Supplementary Tables and Figure [file mmc1.pdf]

Supplementary Material

Supplementary Figure 1: Histogram of distribution of PRS by sex

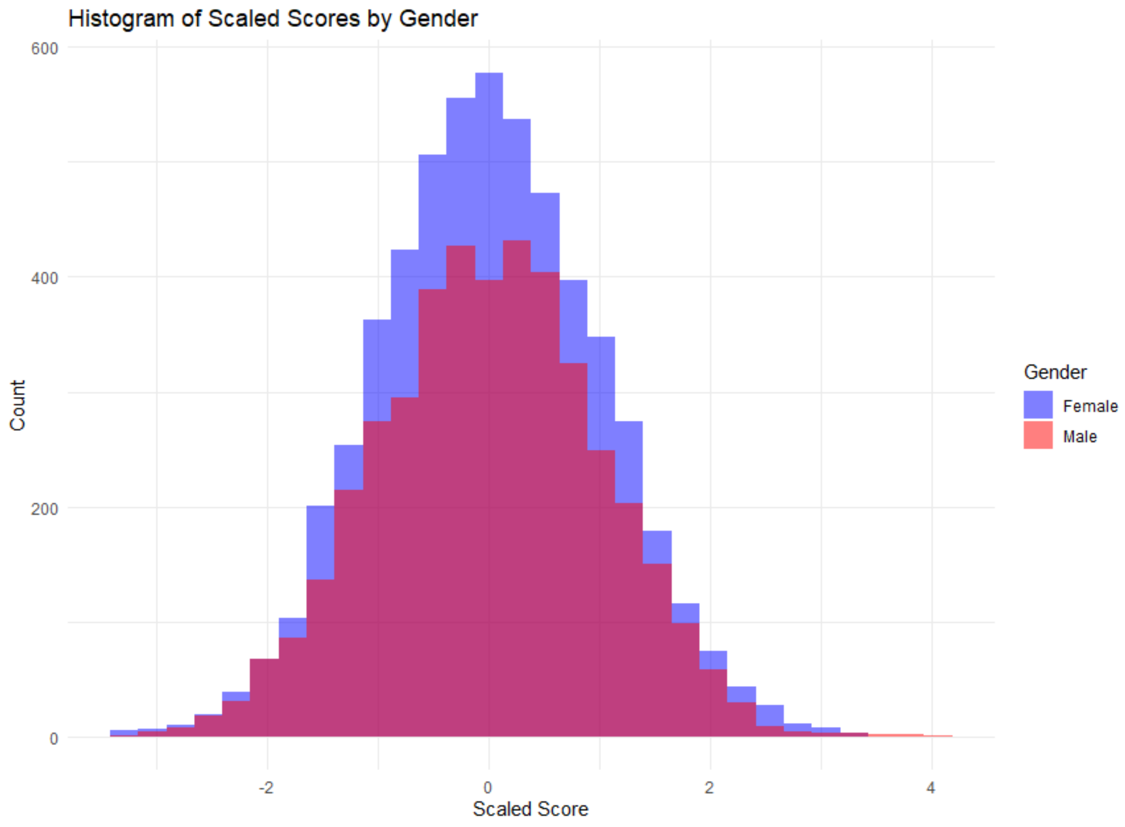

PRS = Polygenic Risk Score

Supplementary Table 1: Poisson regression analyses of associations of optional variables and prevalent CKD on top of current screening criteria in women and men overall and across ethnic groups

	Women			Men		
	PR [95% CI]	AIC	p-value LRT	PR [95% CI]	AIC	p-value LRT
Overall						
Base Model *		4273.0			3419.6	
+ low educational level	1.3 (1.1-1.5)	4265.8	0.001	1.1 (0.9-1.6)	3420.2	0.26
+ obesity	1.5 (1.3-1.7)	4257.3	0.001	1.4 (1.1-1.8)	3412.0	0.002
+ high-risk occupation [1]	1.4 (0.9-2.1)	4272.5	0.11	1.2 (0.9-1.6)	3216.3	0.18
Base Model [Subset] [#]		2631.6			1736. 2	
+ genetic risk factors[#]	1.1 (0.8-1.3)	2633.4	0.63	1.1 (0.8-1.5)	1737.8	0.53
Dutch						
Base Model *		600.3			519.3	
+ low educational level	1.2 (0.4-3.6)	602.4	0.79	2.2 (1-4.4)	518.8	0.07
+ obesity	0.9 (0.4-1.8)	602.3	0.77	1.4 (0.8-2.5)	520.0	0.30
+ high-risk occupation [1]	0	602.1	0.54	1.5 (0.7-3.2)	520.4	0.33

SA Surinamese						
Base Model *		709.5			613.8	
+ low educational level	1.6 (1-2.4)	708.2	0.07	0.8 (0.5-1.3)	615.2	0.43
+ obesity	1.8 (1.2-2.6)	704.0	0.005	1.9 (1.4-2.7)	606.7	0.002
+ high-risk occupation [1]	1.2 (0.5-2.9)	711.4	0.72	1.1 (0.7-1.9)	615.6	0.65
Afr. Surinamese						
Base Model *		1043.1			694.3	
+ low educational level	1.3 (0.7-2.3)	1044.6	0.49	1.2 (0.6-2.3)	696.2	0.70
+ obesity	1.6 (1.1-2.2)	1038.0	0.009	1.5 (1-2.3)	692.9	0.07
+ high-risk occupation [1]	0.8 (0.2-3)	1045.1	0.79	1.3 (0.8-2.2)	695.5	0.37
Ghanaian						
Base Model *		705.5			494.8	
+ low educational level	1.2 (0.8-1.8)	707.4	0.30	1.0 (0.6-1.8)	495.0	0.93
+ obesity	1.1 (0.8-1.6)	707.2	0.61	0.8 (0.4-1.4)	494.2	0.43
+ high-risk occupation [1]	1.0 (0.5-1.9)	707.5	0.95	0.7 (0.2-2.2)	487.7	0.53
Turkish						

Base Model *		585.7			530.2	
+ low educational level	1.0 (0.6-1.7)	587.7	0.89	1.3 (0.8-2.0)	531.2	0.32
+ obesity	1.3 (0.9-2.1)	586.3	0.24	1.1 (0.7-1.7)	532.2	0.81
+ high-risk occupation [1]	1.8 (0.9-3.6)	585.9	0.18	0.7 (0.3-1.5)	531.4	0.38
Moroccan						
Base Model *		622.6			413.8	
+ low educational level	1.1 (0.6-1.8)	624.5	0.81	1.0 (0.6-1.7)	415.8	0.90
+ obesity	1.2 (0.8-1.9)	624.1	0.50	1.9 (1.1-3.1)	411.4	0.04
+ high-risk occupation [1]	3.8 (0.9-15.7)	622.2	0.13	1.5 (0.7-3.3)	414.7	0.29

AIC = Akaike Information Criterion; LRT = Likelihood Ratio Test; * Base model included hypertension, diabetes mellitus, and cardiovascular disease; CI = Confidence Interval; [#]Analyses conducted in a subgroup of the cohort where data from Whole Genome Sequencing was available; [1] High-risk occupation analyses restricted to those that work, additional analyses included a subgroup analysis excluding all those that did not work (AIC Base model: 3366.9 in men; 5378.3 in women)

Supplementary table 2: Prevalence ratios for CKD of the current screening criteria and optional variables included in the final model in women and men

	Women		Men	
	PR (95% CI)	p-value LRT	PR (95% CI)	p-value LRT
Hypertension	1.9 (1.6-2.3)	0.001	4.4 (3.5-5.6)	0.001
Diabetes	1.7 (1.4-2.0)	0.001	2.5 (2.0-3.0)	0.001
CVD	1.4 (1.1-1.7)	0.005	1.1 (0.9-1.5)	0.35
Obesity	1.4 (1.2-1.7)	0.001	1.4 (1.1-1.8)	0.002
Low Education	1.3 (1.1-1.6)	0.01	.*	.
AIC*	4253.2		3412.0	

*Final model did not include *low educational level* for men. LRT= likelihood ratio test, AIC = Akaike Information Criterion

Supplementary Table 3: Discriminative ability and Predictive measures for the base model with current screening criteria and final model with current criteria and optional variables, in women and men overall and across ethnic groups

		Women		Men	
		Base Model	Final Model	Base Model	Final Model
Overall	AUC [95% CI]	0.65 (0.63-0.67)	0.66 (0.64-0.68)	0.75 (0.73-0.77)	0.76 (0.74-0.78)
	Sensitivity	58.3	50.0	80.4	80.4
	Specificity	65.2	77.6	60.8	60.8
	NPV	96.3	96.2	98.1	98.1
	PPV	9.2	11.8	10.7	10.7
Dutch	AUC [95% CI]	0.62 [0.55-0.68]	0.62 [0.56-0.69]	0.75 [0.70-0.80]	0.76 [0.71-0.81]
	Sensitivity	47.7	44.7	83.1	83.1
	Specificity	74.9	77.4	63.8	63.9
	NPV	98.0	97.9	99.1	99.1
	PPV	5.4	5.6	7.2	7.2
SA Surinamese	AUC [95% CI]	0.68 [0.62-0.74]	0.70 [0.64-0.75]	0.81 [0.77-0.85]	0.83 [0.79-0.86]
	Sensitivity	68.6	61.7	89.6	68.8
	Specificity	58.6	72.0	58.5	82.8

	NPV	96.1	96.1	98.3	96.6
	PPV	11.1	14.2	16.6	27.0
Afr. Surinamese	AUC [95% CI]	0.66 [0.62-0.71]	0.68 [0.63-0.72]	0.69 [0.65-0.75]	0.72 [0.66-0.76]
	Sensitivity	75.5	65.7	78.0	79.0
	Specificity	49.9	68.3	52.8	52.0
	NPV	96.9	96.8	97.1	97.1
	PPV	9.0	11.9	10.5	10.5
Ghanaian	AUC [95% CI]	0.57 [0.52-0.63]	0.59 [0.53-0.63]	0.66 [0.60-0.71]	0.66 [0.61-0.72]
	Sensitivity	66.0	71.8	86.3	86.3
	Specificity	47.7	43.1	39.8	39.8
	NPV	93.5	94.0	96.9	96.9
	PPV	11.0	11.0	11.4	11.4
Turkish	AUC [95% CI]	0.60 [0.54-0.67]	0.61 [0.55-0.68]	0.72 [0.66-0.78]	0.72 [0.66-0.78]
	Sensitivity	38.9	37.6	69.0	69.0
	Specificity	79.3	84.7	69.2	69.2
	NPV	95.4	95.6	97.7	97.7
	PPV	10.5	13.3	10.4	10.4
Moroccan	AUC [95% CI]	0.57 [0.52-0.63]	0.56 [0.50-0.63]	0.75 [0.68-0.81]	0.76 [0.69-0.83]
	Sensitivity	39.2	29.1	74.0	72.0
	Specificity	73.5	87.5	68.2	73.5

	NPV	95.5	95.6	98.4	98.4
	PPV	7.6	11.5	8.7	10.1

*Base model included hypertension, diabetes mellitus, and cardiovascular disease; **Final model included hypertension, diabetes mellitus, cardiovascular disease, low education (women only), and obesity; PPV = Positive predictive value; NPV = Negative predictive value; AUC = Area under the curve; CI = Confidence Interval

Supplementary table 4: Poisson regression analyses of optional variables and prevalent CKD on top of current screening criteria in women and men overall and across ethnic groups (Alternative approach)

	Women			Men		
	PR [95% CI]	AIC	p-value LRT	PR [95% CI]	AIC	p-value LRT
Overall						
Base Model*		5661			3614	
+ low educational level	1.4 (1.2-1.7)	5591	0.001	1.3 (1-1.6)	3588	0.04
+ obesity	1.6 (1.4-1.9)	5619	0.001	1.6 (1.3-1.9)	3565	0.001
+ high risk occupation [1]	1.2 (0.5-2.6)	2462	0.68	1.1 (0.7-1.7)	1958	0.65
Base Model * [Subset] [#]		2642			1797	
+ genetic risk factors[#]	1.1 (0.9-1.4)	2643	0.55	1.1 (0.8-1.4)	1798	0.59
Dutch						

Base Model *		632			539	
+ low educational level	0.9 (0.3-3)	633	0.92	2.4 (1.2-4.8)	531	0.04
+ obesity	1 (0.5-1.8)	634	0.89	1.6 (1-2.8)	538	0.09
+ high risk occupation [1]	0	351	0.71	1.3 (0.3-5.1)	305	0.73
SA Surinamese						
Base Model *		840			718	
+ low educational level	1.7 (1.2-2.5)	834	0.01	1 (0.6-1.6)	715	0.97
+ obesity	1.9 (1.3-2.6)	831	0.001	2.2 (1.6-3.1)	705	0.001
+ high risk occupation [1]	0.9 (0.1-6.2)	391	0.94	1.4 (0.6-3.3)	363	0.48
Afr. Surinamese						
Base Model *		1181			751	
+ low educational level	1.4 (0.8-2.4)	1168	0.25	1.3 (0.7-2.5)	750	0.39
+ obesity	1.8 (1.3-2.4)	1169	0.001	1.8 (1.2-2.7)	746	0.007
+ high risk occupation [1]	1.8 (0.5-6.7)	618	0.44	1.3 (0.6-3)	408	0.52
Ghanaian						
Base Model *		853			484	

+ low educational level	1.1 (0.8-1.6)	840	0.46	1 (0.6-1.8)	485	0.90
+ obesity	1.3 (0.9-1.8)	853	0.21	0.9 (0.5-1.6)	486	0.69
+ high risk occupation [1]	0.3 (0-2.3)	400	0.18	0.5 (0.1-1.9)	288	0.24
Turkish						
Base Model *		988			598	
+ low educational level	1.2 (0.9-1.6)	976	0.33	1.4 (0.9-2.2)	592	0.18
+ obesity	1.7 (1.2-2.3)	982	0.004	1.1 (0.7-1.7)	600	0.67
+ high risk occupation [1]	1.3 (0.2-8.6)	308	0.78	0.6 (0.2-1.8)	356	0.31
Moroccan						
Base Model *		1130			497	
+ low educational level	1.3 (0.9-1.7)	1122	0.17	1.5 (0.9-2.5)	495	0.12
+ obesity	1.3 (1-1.7)	1129	0.13	2.7 (1.7-4.4)	480	0.001
+ high risk occupation [1]	10.3 (2.5-43)	375	0.09	2.4 (0.8-7.7)	238	0.20

AIC = Akaike Information Criterion; LRT = Likelihood Ratio Test; * including eligibility (yes/no) based on presence of hypertension or diabetes mellitus or CVD; CI = Confidence Interval; [#]Analyses conducted in a subgroup of the cohort where data from Whole Genome Sequencing was available; [1] High risk occupation analyses restricted to those that work

Supplementary table 5: Discriminative ability and predictive measures for the base model with current screening criteria and final model with current criteria and optional variables, in women and men overall and across ethnic groups (Alternative approach)

		Women		Men	
		Base Model *	Final Model **	Base Model *	Final Model **
Overall	AUC [95% CI]	0.62 [0.59-0.65]	0.59 [0.58-0.61]	0.68 [0.66-0.71]	0.66 [0.64-0.67]
	Sensitivity	66.5	76.6	84.2	88.0
	Specificity	58.1	42.2	53.6	44.0
	NPV	96.3	96.4	98.3	98.4
	PPV	9.6	8.2	9.6	8.4
Dutch	AUC [95% CI]	0.61 [0.56-0.68]	0.60 [0.54-0.66]	0.73 [0.68-0.77]	0.73 [0.68-0.77]
	Sensitivity	49.1	50.7	85.1	88.1
	Specificity	74.3	69.1	61.2	58.3
	NPV	98.0	97.9	99.2	99.3
	PPV	5.4	4.7	6.9	6.6
SA Surinamese	AUC [95% CI]	0.62 [0.56-0.67]	0.61 [0.57-0.65]	0.62 [0.56-0.67]	0.68 [0.65-0.70]
	Sensitivity	76.6	81.8	94.0	94.8
	Specificity	51.1	40.4	48.7	41.6
	NPV	96.6	96.6	98.9	98.9
	PPV	10.9	9.7	14.6	13.2
Afr. Surinamese	AUC [95% CI]	0.62 [0.59-0.65]	0.60 [0.57-0.63]	0.64 [0.60-0.68]	0.63 [0.60-0.66]

	Sensitivity	80.4	86.5	81.9	86.6
	Specificity	43.9	33.1	46.8	40.3
	NPV	97.0	97.2	97.3	97.7
	PPV	9.1	8.3	9.8	9.3
Ghanaian	AUC [95% CI]	0.55 [0.51-0.60]	0.54 [0.51-0.57]	0.61 [0.56-0.65]	0.59 [0.55-0.63]
	Sensitivity	68.0	86.4	87.1	90.0
	Specificity	42.9	22.2	35.1	28.6
	NPV	93.2	94.4	97.0	97.1
	PPV	10.4	9.7	10.3	9.7
Turkish	AUC [95% CI]	0.59 [0.55-0.64]	0.54 [0.50-0.58]	0.68 [0.63-0.73]	0.63 [0.60-0.67]
	Sensitivity	59.1	72.6	78.5	88.6
	Specificity	60.0	34.6	57.9	38.4
	NPV	95.1	94.3	98.1	98.5
	PPV	10.0	7.8	8.8	6.9
Moroccan	AUC [95% CI]	0.62 [0.58-0.66]	0.56 [0.52-0.60]	0.66 [0.60-0.72]	0.61 [0.55-0.65]
	Sensitivity	57.1	69.7	72.6	79.0
	Specificity	67.7	42.1	60.1	42.3
	NPV	95.8	95.2	98.1	97.8
	PPV	11.1	7.8	7.3	5.6

* Base model included eligibility (yes/no) based on presence of hypertension or diabetes mellitus or cardiovascular disease; **Final model included eligibility, low education (for women only), and obesity; PPV = Positive predictive value; NPV = Negative predictive value; AUC = Area under the curve; CI = Confidence Interval; SA Surinamese = South-Asian Surinamese
